# *Bacteroides fragilis* derived metabolites, identified by molecular networking, decrease *Salmonella* virulence in mice model

**DOI:** 10.3389/fmicb.2022.1023315

**Published:** 2022-11-10

**Authors:** Thomas Gautier, Nolwenn Oliviero, Solenn Ferron, Pierre Le Pogam, Sandrine David-Le Gall, Aurélie Sauvager, Patricia Leroyer, Isabelle Cannie, Sarah Dion, Alaa Sweidan, Olivier Loréal, Sophie Tomasi, Latifa Bousarghin

**Affiliations:** ^1^INSERM, Univ Rennes, INRAE, UMR 1241, Nutrition Metabolisms and Cancer Institute, Rennes, France; ^2^Univ Rennes, CNRS, ISCR (Institut des Sciences Chimiques de Rennes) - UMR 6226, Rennes, France; ^3^BioCIS, Université Paris-Sud, CNRS, Université Paris-Saclay, Châtenay-Malabry, France; ^4^Univ Rennes, INSERM, EHESP, Irset (Institut de recherche en santé, environnement et travail) – UMR_S 1085, Rennes, France; ^5^Laboratory of Microbiology, Department of Life and Earth Sciences, Faculty of Sciences I, Lebanese University, Hadath Campus, Beirut, Lebanon

**Keywords:** *Bacteroides fragilis*, supernatant, fractionation, microbiota, molecular networking, metabolites, cholic acid, virulence factors

## Abstract

In the gut microbiota, resident bacteria prevent pathogens infection by producing specific metabolites. Among bacteria belonging to phylum *Bacteroidota*, we have previously shown that *Bacteroides fragilis* or its cell-free supernatant inhibited *in vitro Salmonella* Heidelberg translocation. In the present study, we have analyzed this supernatant to identify bioactive molecules after extraction and subsequent fractionation using a semi-preparative reversed-phase Liquid Chromatography High-Resolution Tandem Mass Spectrometry (LC-HRMS/MS). The results indicated that only two fractions (F3 and F4) strongly inhibited *S.* Heidelberg translocation in a model mimicking the intestinal epithelium. The efficiency of the bioactive fractions was evaluated in BALB/c mice, and the results showed a decrease of *S.* Heidelberg in Peyer’s patches and spleen, associated with a decrease in inflammatory cytokines and neutrophils infiltration. The reduction of the genus *Alistipes* in mice receiving the fractions could be related to the anti-inflammatory effects of bioactive fractions. Furthermore, these bioactive fractions did not alter the gut microbiota diversity in mice. To further characterize the compounds present in these bioactive fractions, Liquid Chromatography High-Resolution Tandem Mass Spectrometry (LC-HRMS/MS) data were analyzed through molecular networking, highlighting cholic acid (CA) and deoxycholic acid. *In vitro*, CA had inhibitory activity against the translocation of *S.* Heidelberg by significantly decreasing the expression of *Salmonella* virulence genes such as *sipA*. The bioactive fractions also significantly downregulated the flagellar gene *fliC*, suggesting the involvement of other active molecules. This study showed the interest to characterize better the metabolites produced by *B. fragilis* to make them means of fighting pathogenic bacteria by targeting their virulence factor without modifying the gut microbiota.

## Introduction

Among *Salmonella, Salmonella* Heidelberg (*S*. Heidelberg), causing severe extra-intestinal infections, displays a hypermutator phenotype related to the frequent occurrence of mutations in the genes involved in the methyl mismatch repair system (Wilmshurst and Sutcliffe, 1995; Le Gall et al., 2009; Le Gall-David et al., 2015). These bacteria have adapted to adverse and stringent environmental conditions, including the pressure of antibiotic exposure (Blázquez, 2003). New therapeutic alternatives, such as next-generation probiotics, are being developed to treat them. *Bacteroides fragilis* (*B. fragilis*), a part of commensal gastrointestinal flora, an obligate anaerobe, is proposed because of its well-known beneficial properties in food metabolism, in gut ecology and in anti-inflammatory effects (Wexler, 2007; Chu et al., 2016; Deng et al., 2016; Wang et al., 2017; Díaz-Garrido et al., 2021). It was also shown that *B. fragilis* and its cell-free supernatant inhibited the translocation of *S.* Heidelberg, in a complex multicellular model mimicking the intestinal epithelium (Vernay et al., 2020). *Salmonella* pathogenicity depends on a variety of virulence factors, including flagella (FliC) and three secretion systems (T3SSs). FliC enables *Salmonella* to penetrate the gastrointestinal mucus layer by triggering rapid and efficient contact with the cells lining the epithelium. T3SS system is a molecular syringe that can translocate the effector proteins such as SipA, SipC, SopA, SopB, and SopE2 directly from the bacteria into the cytosol of cells where they subvert cellular processes that contribute to bacterial invasion, actin rearrangement, membrane ruffling and other aspects of virulence (Hayward and Koronakis, 2002). SipA effector, actin-binding protein, directly modulate host actin dynamics to facilitate bacterial uptake (Zhang et al., 2018).

Our previous results suggested that *B. fragilis* produces bioactive molecules against *S.* Heidelberg virulence in its cell-free supernatant (Vernay et al., 2020). In addition, several probiotics and commensal bacteria, including *Lactobacillus* GG (LGG) and *Bifidobacterium breve*, have been shown to secrete metabolites exhibiting a beneficial effect against several intestinal disorders (traveler’s diarrhea, antibiotic-associated diarrhea, and acute infectious diarrhea; Reid et al., 2003).

Numerous studies have shown that the gut microbiota contains healthy bacteria secreting metabolites that could help to fight diseases in the gut and other organs (Antunes et al., 2014; Postler and Ghosh, 2017; Sittipo et al., 2019). Accordingly, the intestinal microbiota generates metabolites that include folate, indoles, *gamma*-aminobutyric acid, serotonin, secondary bile acids (BAs), desaminotyrosine, and short-chain fatty acids (SCFAs). Several studies have suggested that these microbiota-derived metabolites, present in cell-free supernatants, may be an alternative to antibiotics in the struggle against pathogens such as *Escherichia coli* and *Salmonella* (Antunes et al., 2014; Li et al., 2018; Piqué et al., 2019; Hove et al., 2020). Peixoto et al. (2017) have shown that small molecules from human fecal microbiome such as aromatic compounds inhibited invasion of *Salmonella* SL1344 to Hela cells (Peixoto et al., 2017).

In the present study, we have characterized the cell-free supernatant of *B. fragilis* by fractionating it and assessed its ability to inhibit *Salmonella* translocation. The effectiveness of the bioactive fractions was also investigated in a mouse model. A molecular networking analysis of the bioactive fractions was carried out to identify bioactive molecules.

## Materials and methods

### Bacteria and growth conditions

*Bacteroides fragilis* (*B. fragilis*) NTBF (Non Toxigenic *Bacteroides fragilis*), ATCC 25285 (Sears et al., 2014; Choi et al., 2016), was purchased from the American Type Culture Collection. *Bacteroides fragilis* growth conditions were described in a previous study (Vernay et al., 2020). The supernatant was filtered through a 0.22 μm-pore-size syringe filter as we described in Vernay et al., 2020.

*Salmonella* Heidelberg B182 strain (*S*. Heidelberg) was isolated on LB (Luria-Bertani) agar and grown in LB medium overnight at 37°C. *S.* Heidelberg was then subcultured by dilution in LB medium followed by incubation for 90 min at 37°C (Le Bars et al., 2012).

### *Bacteroides fragilis* supernatant extraction

*Bacteroides fragilis* was cultured in 500 ml of complete DMEM (Dulbecco’s Modified Eagle Medium) containing 20% of fetal bovine serum (FBS) and 1% of glutamine in an anaerobic chamber for 24 h. After this incubation, *B. fragilis* culture was centrifuged for 10 min at 11500 x g. Cell-free supernatant was filtered, extracted with 2 × 250 ml of ethyl acetate, and dried under vacuum.

### Supernatant fractionation by semi-preparative HPLC

The supernatant extract was then fractionated by semi-preparative HPLC-DAD (Shimadzu, Marne La Vallée, France) using a reversed-phase Prevail™ C18-Select column (Grace; 250 × 10 mm, 5 μm) and ultrapure water with 0.1% formic acid as solvent A and acetonitrile with 0.1% formic acid as solvent B. The following gradient system was applied at a flow rate of 2 ml/min in the HPLC system: initial: 100% (A); from 0 to 5 min: 100% (A); from 5 to 45 min: 100% (A) to 0% (A); from 45 to 50 min: 0% (A); from 50 to 55 min: 0% (A) to 100% (A); from 55 to 70 min: 100% (A).

The following fractions were collected and evaporated under vacuum: F1 (Fraction 1): rt. = 5–19 min, m = 14.6 mg; F2a: rt. = 19–26 min, m = 4.4 mg; F2b: rt. = 26–31 min, m = 2.7 mg; F3: rt. = 31–38 min, m = 4 mg; F4: rt. = 38–48 min, m = 4.3 mg; F5: rt. = 48–60 min, m = 4.9 mg. Each fraction was then dissolved in MeOH at 1 mg/ml for further analysis.

### *Salmonella* Heidelberg translocation inhibition assay *in vitro*

To evaluate the impact of the selected fractions on *S*. Heidelberg translocation, we used the same model composed of several cells, mimicking the intestinal epithelium, described in Vernay et al., 2020. Briefly, Caco-2/HT29-MTX were seeded on the apical chamber of polycarbonate Transwell® inserts. After 14 days of culture, Raji B cells were added to the basolateral chamber to induce differentiation of Caco-2 cells into M cells for 7 days.

To mimic *Salmonella* infection, *S*. Heidelberg was cultured as described in Vernay et al., 2020 and was added to the apical compartment at a multiplicity of infection (MOI) of 10 bacteria/cells at 37°C for 3 h. After incubation, basal and apical media were collected separately, and colony-forming units were counted. *S*. Heidelberg colonies were enumerated on GTS plates. Results were expressed as a ratio of the number of translocated bacteria collected in the basal compartment and the number of bacteria counted in the apical compartment.

To evaluate the impact of fractions on this *S*. Heidelberg translocation, each fraction (0.1 mg/ml) were added in the apical compartment along with *Salmonella* (MOI of 10). After 3 h of incubation, *S*. Heidelberg was enumerated as described above.

### Bioactive fractions efficiency in inhibiting *Salmonella* Heidelberg translocation in mice model

BALB/c 8-week-old female mice (*n* = 5 per group) were purchased from Janvier (France) and were housed in a specific animal facility with a controlled temperature-humidity. Upon delivery, mice underwent a one-week acclimation period. At the end of the adaptation, animals were randomly divided in the cages into four groups. The first group, control mice, received culture medium, complete DMEM, by gavage. The second group, *Salmonella-infected* mice, received 10^8^ colony forming unit (CFU) of *S*. Heidelberg diluted in complete DMEM. The third group received 10^8^ CFU of *S*. Heidelberg and bioactive fractions (mixture of F3 and F4 (1:1, v/v) corresponding to 0.1 mg/ml), and the last control group received only bioactive fractions (mixture of F3 and F4) diluted in complete DMEM. At 2, 4, and 7 days of treatments, DMEM was re-administered in the first and the second group, and bioactive fractions were re-administered in the last two groups. After 8 days of treatment, mice were euthanized before samples collection.

All experimental protocols were approved by the Adaptive Therapeutics Animal Care and Use Committee (APAFIS#31484–2,021,050,308,355,787 v6). Experiments were monitored in the ARCHE-BIOSIT animal lab at Rennes University and were carried out under level 2 high-risk biosecurity procedures.

### *Salmonella* Heidelberg dissemination in spleen, Peyer’s patches, and liver

Bacterial loads in feces, colon, spleen, Payer’s patches and liver were removed by vortexing with 3 mm glass beads and gravity sedimentation for 10 min. They were then serially diluted in water and spread on LB rapid’ *Salmonella* chromogenic agar (Biorad, France) to identify only *Salmonella* colonies; the results represented only these enumerated colonies.

### RNA extraction, cDNA synthesis, RT-qPCR

For *in vitro* samples, after incubating the multicellular model with *S*. Heidelberg for 3 h, 350 μl of lysis buffer was added until 20 min at ambient temperature to recover cells’ RNA. Then, 50 μl of lysozyme solution (20 mg/ml) was added for 30 min to recover the bacterial RNA.

For the *in vivo* assay, the mice colon was collected and frozen in liquid nitrogen and then stored at −80°C for later.

RNAs were extracted using an RNA purification kit, including DNAse digestion on-column (MACHEREY-NAGEL), following manufacturer instructions. RNAs were transcribed into cDNA with High Capacity cDNA Reverse Transcription Kit (Applied Biosystems) and, following the protocol provided by the manufacturer. The no reverse transcriptase controls were also set up along with sample reactions so as to confirm the absence of genomic DNA contamination. Then, the selected genes were relatively quantified using StepOnePlus (Applied Biosystems) with SYBR Green PCR Master Mix (Applied Biosystems; Le Bars et al., 2012). All primers used in this study are described in Table 1. Data analysis was carried out with QuantStudio™ Real-Time PCR Software. Ct values for each gene were normalized to housekeeping gene Ct values: *16S* RNA for *S*. Heidelberg and *hprt1* (hypoxanthine phosphoribosyltransferase 1) for mice.

**Table 1:** Primers used in this study.

Genes	Forward	Reverse
*16S RNA*	AGGCCTTCGGGTTGTAAAGT	GACTCAAGCCTGCCAGTTTC
*sipA*	AAGATTTTCCCGTGATC	GCTTTCTTAGCGACATT
*fliC*	TCTTCCGGTCTGCGTATCAA	CCTTTGATGTTCGCGGTGAA
*cldn-1*	AAGGTGCTGCTGAGGGTAGA	GGTGTTGGGTAAGAGGTT GT
*cldn-2*	AAGGTGCTGCTGAGGGTAGA	AGTGGCAGAGATGGGATTTG
*occludin*	AGGCTTCTGGATCTATGTACG	ATGAACCCCAGGACAATG
*muc-2*	TGTGGAACCGGGAAGATG	GACCACAGGTATGGTTCTGGA
*hpr1*	CTCATGGACTGATTATGGACAGGAC	GCAGGTCAGCAAAGAACTTATAGCC

### Plasma cytokine measurement

Blood samples were obtained at the time of sacrifice *via* cardiac puncture, and they were collected in heparin tubes. After centrifugation, the plasma was diluted, and cytokines were quantified. To investigate these murine cytokines, bead-based immunoassays were used, according to the manufacturer’s protocol, with a filter plate and a vacuum filtration system for washing steps (LEGENDplex™ Mouse Inflammation Panel kit, BioLegend). Samples were analyzed on an LSR Fortessa X-20 (Becton Dickinson, Plateform CytomrTRI, SFR Biosit–UMS CNRS 3480-INSERM 018, Rennes). The concentrations of these cytokines in plasma were calculated based on their corresponding standard curves.

### Histology and neutrophils infiltration score

Tissue samples from the colon were collected and stored in 4% paraformaldehyde solution for 24 h and then included in kerosene wax with Excelsior ES 50 for H&E staining. Kerosene block sections were cut to a 4 μm thickness and mounted on glass slides where longitudinal histological sections were observed. Tissue sections were stained with Hematoxylin / Eosin / Saffron with Leica ASP 300. Colon images were obtained using digital slide scanner Nanozoomer 2.0RS and analyzed with the NDP.view2 viewing software. The morphology of the colon sections was observed to detect neutrophils infiltration and the percentage of neutrophils infiltration was calculated by considering the surface area of the infiltrate zone compared to the whole colon section. The infiltration rate is calculated taking into account the surface of the infiltrate on the total surface.

### Fecal microbiota analysis using 16S rDNA

Fecal samples were collected on day 8 and were immediately stored at −80°C for DNA extraction using the Qiagen Powerfecal Pro DNA kit. V3-V4 region of the 16S rDNA was amplified using forward primers containing the sequence (CTTTCCCTACACGACGCTCTTCCGATCTTACGGRAGGCAGCAG) and R784 reverse primers (GGAGTTCAGACGTGTGCTCTTCCGATCTTACCAGGGTATCTAATCCT). To reveal 510 bp amplicons by electrophoresis, PCR was conducted using 30 amplification cycles and an annealing temperature of 62°C. Single multiplexing was performed using a 6-bp internal index, which was added to R784 in a second 12-cycle PCR using the forward primer (AATGATACGGCGACCACCGAGATCTACACTCTTTCCCTACGAC) and reverse primer (CAAGCAGAAGACGGCATACGAGAT-index-GTGACTGGAGTTCAGACGTGT). Purified PCR products were placed into the Illumina MiSeq automated system. The Illumina V3 kit enables 300 bp paired-end reads. Thus, reads from two ends limited by the two primers could be assembled, generating high-quality reads from all targeted regions. Each pair-end sequence was assembled using Flash v1.2.6 software. Qiime 2 pipeline was used to process raw sequences (v. 2018.4).^1^ Files were imported in “PairedEndFastqManifestPhred33” format; Dada 2 was performed to denoising reads (Callahan et al., 2016). Reverse and Forward sequences were truncated to 240 bases, and all other parameters were set to default. Detection of the chimeric sequence was provided by VSEARCH (Rognes et al., 2016). Silva (version 138) reference database (Quast et al., 2012) was used to identify non-16S rDNA genes, and for open reference clustering, with a similarity threshold of 99%, of amplicon sequence variants (ASVs). ASVs present in a single sample or those with total frequency of less than 10 were removed. Reads that were unassigned or assigned to Archaea were removed from the final table. QIIME2 diversity-metrics-phylogenetic plugin was performed to Core diversity analysis, with a specific sampling depth of 10,950 reads.

### LC–MS/MS analysis

Complete DMEM, supernatant and all fractions (F1, F2a, F2b, F3, F4, and F5) were analyzed using an Agilent 6,530 Accurate-Mass Q-TOF hyphenated with a 1,260 Agilent Infinity LC system fitted with a Sunfire Analytical C18 column (150 × 2.1 mm, i. d. 3.5 μm, Waters). The MS system was operating in either positive or negative polarity. HPLC analyses were performed by gradient elution based on the following program: A (0.1% formic acid in Milli-Q water) and B (acetonitrile); T, 0–2 min, 0% B; 2–30 min, 100% B linear; 30–40 min, 100% B; 40–50 min, 0% B with a flow rate of 0.25 ml/min. ESI (electrospray ionization) conditions were set as follows: capillary temperature at 320°C, source voltage at 3500 V in positive-ion mode and 2,500 V in negative-ion mode, and a sheath gas flow rate at 10 l/min. Fragmentor and skimmer voltages were set at 175 and 65 V, respectively. The injected volume was 8 μl. Mass spectrometric analyses were divided into six scan events using a data-dependent scanning method: at first, positive or negative MS (mass range: *m/z* 100 to 1,200), and data-dependent MS/MS scans of the five most intense ions, from the first scan event at three collision energies (*viz*. 30, 50, and 70 eV, in positive-ion mode and 10, 25 and 40 eV in negative polarity). Only singly charged species could be selected for subsequent fragmentation with an isolation of *m/z* 1.3 a.m.u. To perform the real-time lock mass correction, a solution including trifluoroacetic acid (CF_3_CO_2_H, *m/z* 112.98559 in negative-ion mode), purine (C_5_H_4_N_4_ at *m/z* 121.050873 in positive polarity) and hexakis (1*H*, 1*H*, 3*H*-tetrafluoropentoxy)-phosphorene (C_18_H_18_O_6_N_3_P_3_F_24_ at *m/z* 922.009798 (positive), 1033.988109 (negative, trifluoroacetate adduct)) were continuously infused. A permanent MS/MS exclusion list comprising these different reference masses was defined to preclude their selection as precursors for fragmentation.

### Global Natural Products Social Molecular Networking (GNPS) and cytoscape analysis

The MS^2^ data files related to the supernatant and different fractions thereof were converted from the .d (Agilent) standard data format to .mzXML format using the MSConvert software, part of the ProteoWizard package (Chambers et al., 2012). A molecular network was created using the online Molecular Networking workflow (version release_8) at GNPS (Wang et al., 2016)^2^ with a parent mass tolerance of 0.02 Da and an MS/MS fragment ion tolerance of 0.02 Da. A network was then created where edges were filtered to have a cosine score above 0.7 and more than 6 matched peaks. Further edges between two nodes were kept in the network if and only if each of the nodes appeared in each other’s respective top 10 most similar nodes. The spectra in the network were also searched against GNPS spectral libraries. All matches kept between network spectra, and library spectra were required to have a score above 0.7 and at least 3 matched peaks. The molecular networking data were analyzed and visualized using Cytoscape (ver. 3.8.0; Shannon et al., 2003). The different ions were tagged according to their occurrence within the different analyzed samples. Culture medium without bacteria (complete DMEM), supernatant, and each fraction were compared.

### Cholic acid (CA) quantification in bioactive fractions

CA present in the fractions was detected using an analytical HPLC system Diode Array Detector (LC-DAD; Shimadzu, Marne La Vallee, France) associated to an evaporative light scattering detector (ELSD) and quantified by comparison to known amounts of standard CA purchased from Merck. A reversed Hypersil GOLD aQ column (5 μm, 250 × 4.6 mm, ThermoFisher Scientific) was used and a gradient system was applied: A (0.1% formic acid in water) and B (0.1% formic acid in acetonitrile). The following gradient was applied at a flow rate of 0.8 ml/min in the HPLC system: initial: 100% (A); from 0 to 5 min: 100% (A); from 5 to 35 min: 100% (A)/0% (B) to 0% (A)/100% (B); from 35 to 45 min: 100% B; from 45 to 50 min: 100% (A)/0% (B) to 0% (A)/100% (B); from 50 to 55 min: 100% (A). Forty microliters of samples at 1 mg/ml in acetone were injected. A series of standard solutions of synthetic CA with the concentration range 0.005–0.25 mg/ml were prepared. The correlation coefficient of the calibration curve was 0.9976.

### *Salmonella* Heidelberg translocation inhibition assay in the presence of CA

To evaluate the impact of CA on *S*. Heidelberg translocation, synthetic CA (156 μmol/l) diluted in complete DMEM, was added in the apical compartment at the same time as *Salmonella* (MOI of 10). *Salmonella* alone or CA alone were used as controls. After 3 h of incubation, *S*. Heidelberg in the apical and basal compartment was enumerated as described above to determine the rate of translocation.

### Statistics analysis

All experiments were performed in triplicates at least or more for *in vitro* tests. Normality of data distribution was confirmed using the Shapiro–Wilk test, differences within and between groups were calculated using a two-way analysis of variance (ANOVA one way); otherwise, the non-parametric Kruskal-Wallis test was used. PERMANOVA test was used for Beta diversity analysis. Data presented as mean ± SEM, and a value of *p* less than 0.05 was considered significant. The analysis of data and generation of the graph were performed by Graph Pad Prism software (Version 8.0).

## Results

### Two fractions (F3 and F4) of *Bacteroides fragilis* supernatant inhibited *S*. Heildeberg translocation *in vitro*

**Figure 1:**
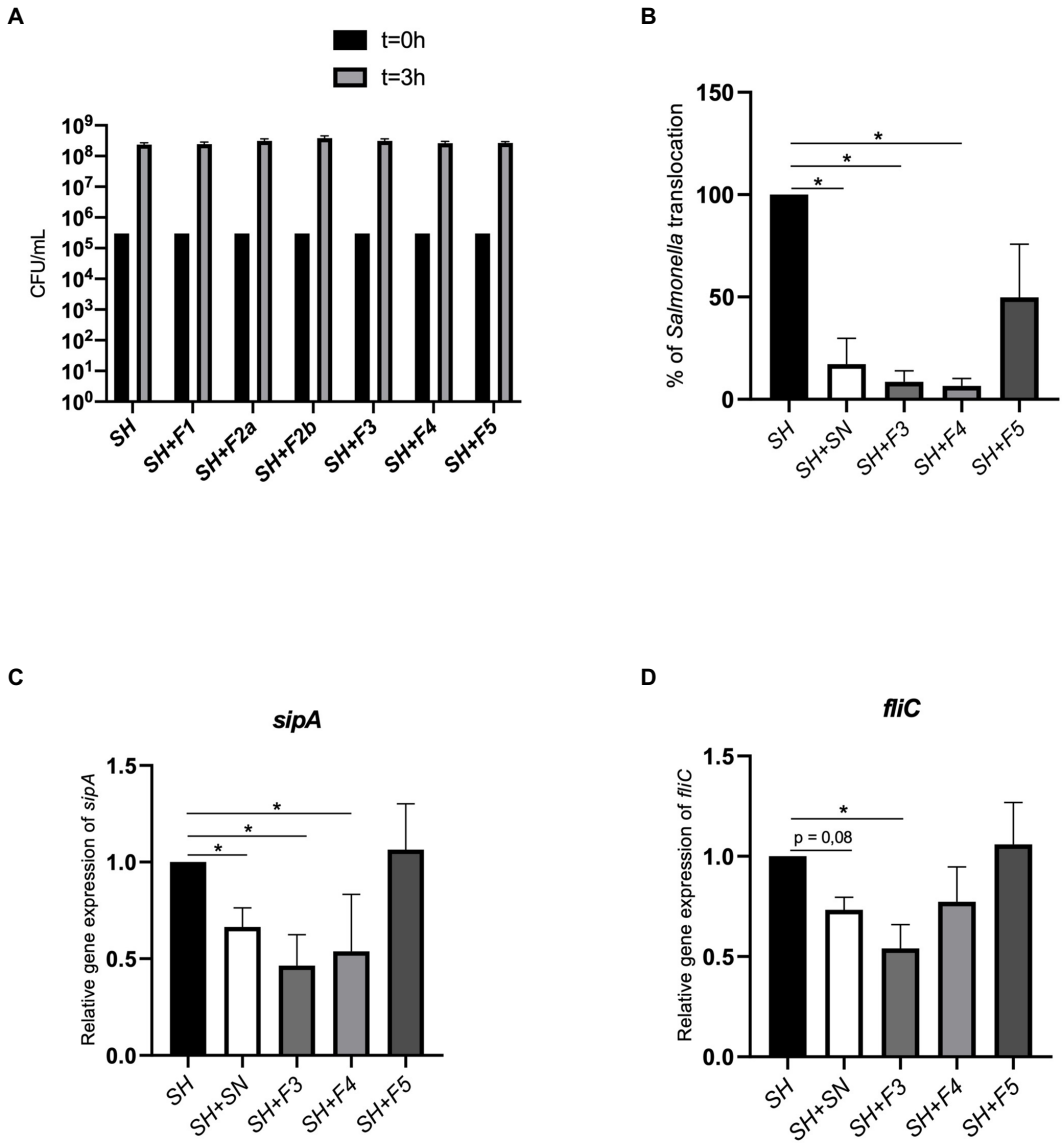
F3 and F4 fractions inhibited *Salmonella* translocation *in vitro*. **(A)** Effect of fractions (F1, F2a, F2b, F3, F4, and F5) on *Salmonella* Heildelberg (SH) growth [colony forming unit (CFU)/ml] after 3 h of infection. **(B)** Impact of *Bacteroides fragilis* F3, F4, or F5 fractions and supernatant (SN) on *Salmonella* Heidelberg translocation across an *in vitro* multicellular model mimicking the intestinal epithelium. Results were expressed as % of translocation normalized to *Salmonella* alone (SH). **(C)** Impact of SN, F3, F4, and F5 on *SipA* expression, **(D)** Impact of SN, F3, F4, and F5 on *fliC* expression. Results were normalized to the cells treated only with *Salmonella*. The Shapiro and the Kruskall-Wallis tests were performed on *n* = 5 for this experiment. The Shapiro test was used to verify normality; the one-way ANOVA test was performed on *n* = 5. All data are presented as means (SEM); *p < 0.05, **p < 0.01.

To facilitate the identification of compounds from the cell-free supernatant of *B*. *fragilis* (SN) involved in *Salmonella* translocation inhibition, a fractionation was performed by a semi-preparative reversed-phase High-Performance Liquid Chromatography (HPLC-DAD). Six fractions (F1, F2a, F2b, F3, F4, and F5) were collected based on the range of retention time (RT in min) and the peak patterns. The ability of the SN and each fraction to inhibit *S*. Heidelberg translocation was evaluated by incubating them on a multicellular model (enterocytes, mucus, and M cells) in the presence of *S*. Heidelberg. After 3 h of incubation with *S*. Heidelberg, the results showed that the F3 and F4 fractions, as well as SN, induced a significant decrease in *S.* Heidelberg translocation (Figure 1B), whereas F5 did not significantly decrease this translocation as for the other fractions (F1, F2a and F2b; data not shown). In addition, these fractions (F1, F2a, F2b, F3, F4, and F5) did not impact *Salmonella* growth (Figure 1A).

To better characterize the mechanism of action of these two fractions, the expression of *Salmonella* virulence genes, *sipA*, and *fliC* was evaluated. The results showed that SN, F3 and F4 significantly decreased *sipA* expression (0.65 ± 0.2, 0.46 ± 0.2 and 0.69 ± 0.2 folds, respectively; Figures 1C,D). Expression of *fliC* was also significantly decreased in the presence of F3 but not with F4 and SN (0.5 ± 0.2 and 0.75 ± 0.2, respectively). F5 did not decrease *sipA* and *fliC* expression (1.06 ± 0.2 and 1.07 ± 0.2, respectively). These results suggested that translocation inhibition in the presence of SN, F3 and F4 could be induced by the decrease of *sipA* and/or *fliC*.

### The mixture of F3 and F4 inhibited *salmonella* translocation in BALB/c mice

**Figure 2:**
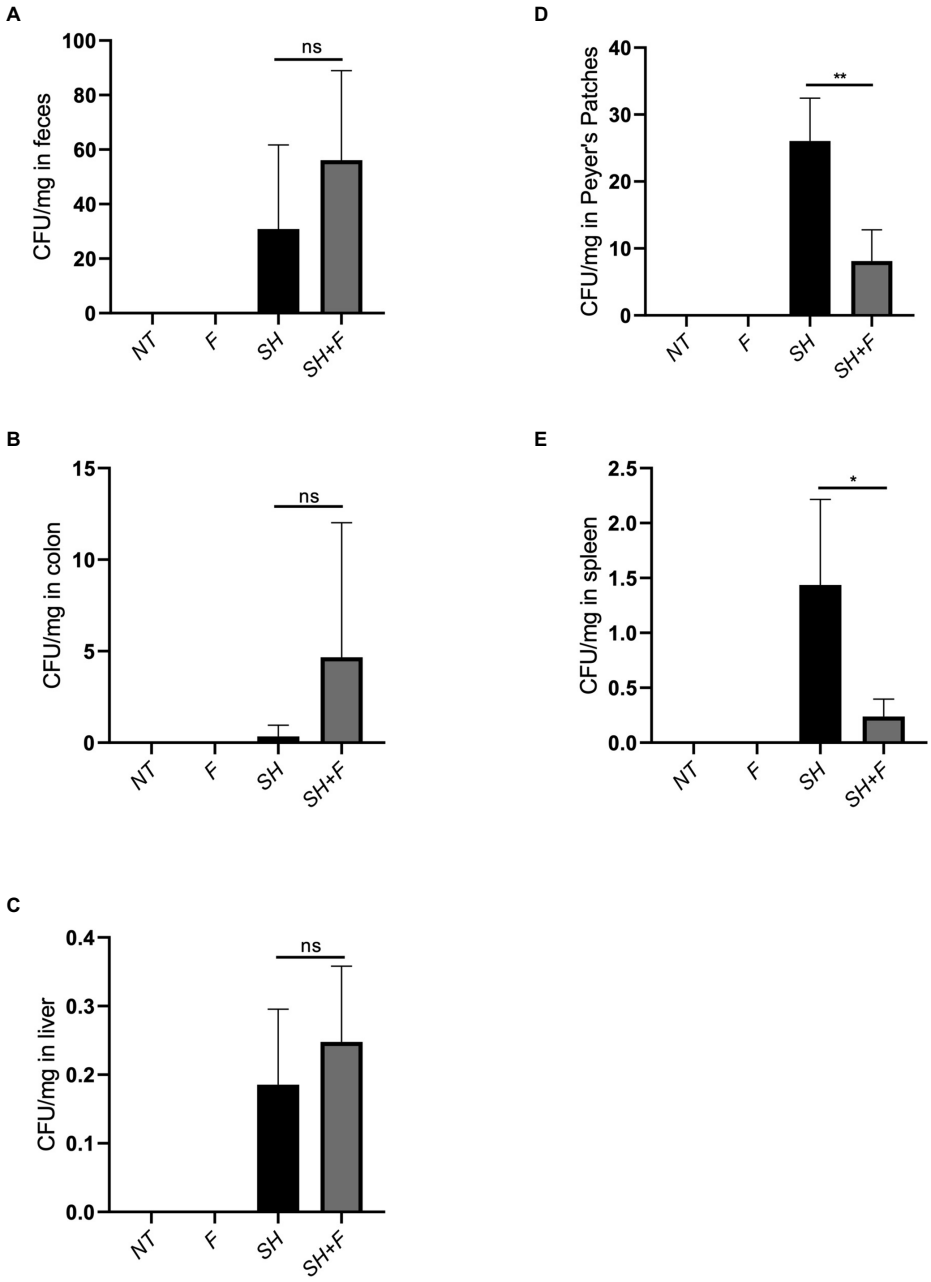
The mixture of F3 and F4 decreased translocation of *S*. Heidelberg in BALB/C mice. Effects of bioactive fractions on bacterial load during *Salmonella* infection. Several groups were used in the study, untreated mice (NT), mice receiving only *Salmonella* (SH), mice receiving only fractions (F) and mice receiving both *Salmonella* and fractions (SH + F). After 8 days of infection, *Salmonella* was extracted from **(A)** feces, **(B)** colon, **(C)** liver, **(D)** Peyer’s patches, and **(E)** spleen, and plated on rapid’*Salmonella* chromogenic agar. CFU/mg represented the number of CFU of bacteria recovered per milligram of tissue. The Shapiro test was used to verify Normality. Statistical data were evaluated by the one-way ANOVA test on *n* = 5. Data were represented by SEM bars, *p < 0.05, **p < 0.01 and ns = no significant.

To determine if the antagonistic activity of bioactive fractions *in vitro* against *S*. Heidelberg could also occur *in vivo*, *S*. Heidelberg was administered orally to mice. For this purpose, the bioactive fractions F3 and F4 were mixed (1:1, v/v) to test their combined effects *in vivo*. After 8 days of *S*. Heidelberg infection, mice were sacrificed, and samples were collected. Feces, colon, liver, Peyer’s patches and spleen were crushed, and numeration of *S*. Heidelberg colonies was performed in each organ. The results showed that there was no significant difference in enumeration in feces, colon, and liver between mice infected with *S*. Heidelberg alone or those having received both *S*. Heidelberg and bioactive fractions of *B*. *fragilis* (Figures 2A–C). However, when fractions were administered with *S*. Heidelberg, a significant two-fold decrease was noted for the number of *S*. Heidelberg in Peyer’s (Figure 2D). In the presence of bioactive fractions, *S*. Heidelberg was also significantly decreased in the spleen compared to mice receiving *S*. Heidelberg alone (Figure 2E). These results suggest that molecules present in the fractions inhibited translocation into Peyer’s patches and then into the spleen.

### Bioactive fractions decreased inflammation during *Salmonella* Heidelberg infection in mice

**Figure 3:**
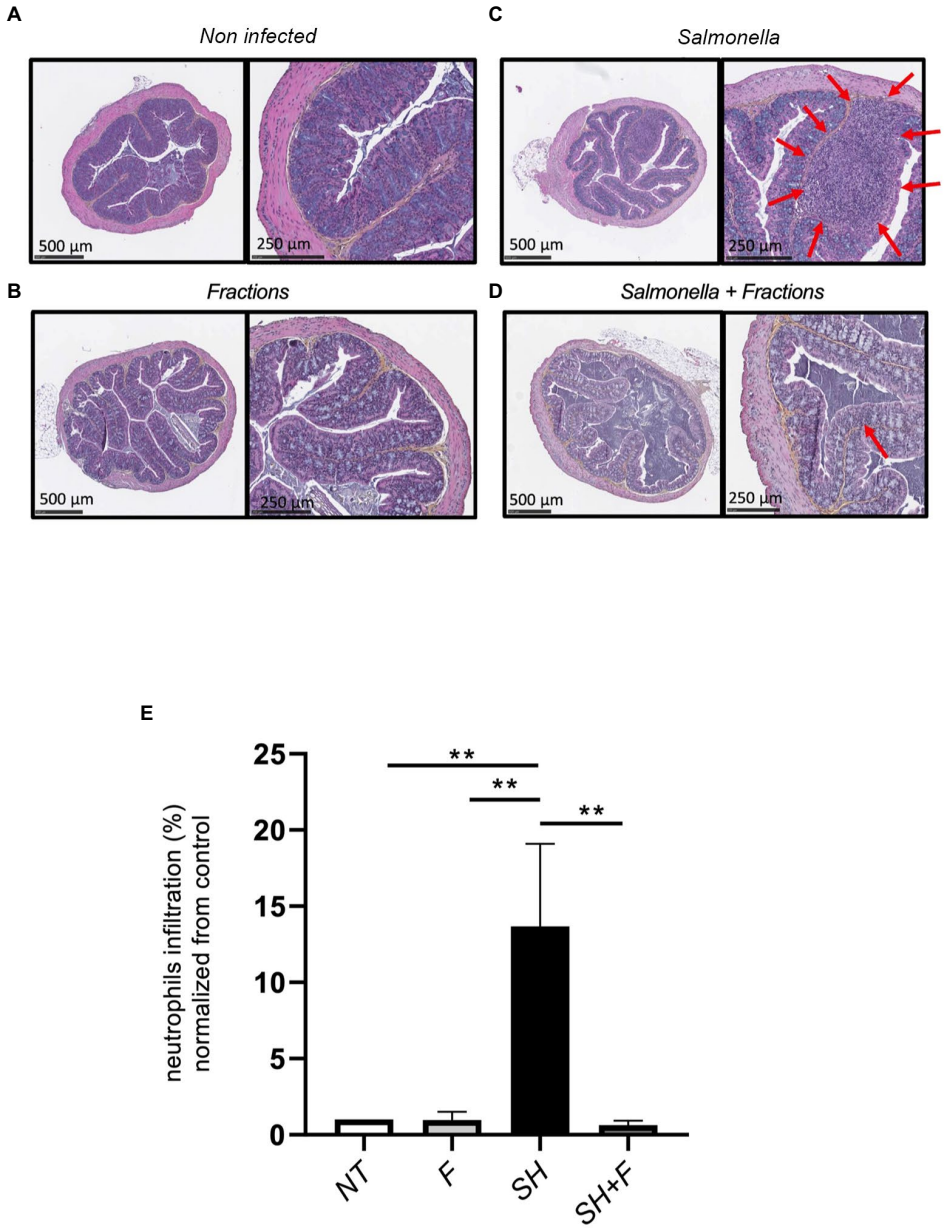
Bioactive fractions inhibited neutrophils infiltration in mice colon. **(A)** Histologic analysis of the colon of untreated mice (NT), **(B)** mice treated by bioactive fractions alone (F), **(C)** mice infected by *Salmonella* Heidelberg alone (SH), and **(D)** mice receiving *Salmonella* and bioactive fractions (SH + F). H&E stained cross-section of the colon of mice of each group showed the presence of neutrophils infiltration (red arrows) in *Salmonella* group only, which was decreased when the bioactive fractions were administered. **(E)** Quantification of neutrophils was represented by % of neutrophils infiltration normalized from the control. Scale bar: 500 and 250 μm. ANOVA one-way statistical analysis test was used for *n* = 5, normality of data was evaluated by Shapiro test. Data were represented by SEM bars **p < 0.01.

Hematoxylin/Eosin (H&E) histology showed that the neutrophils infiltration score was very low (basal level 1%) in mice without bacteria (Figure 3A) or with bioactive fractions (Figure 3B), and no difference was found between the two groups (Figure 3E). In mice receiving only *S*. Heidelberg, infiltration of inflammatory cells can be observed as indicated by the red arrows (Figure 3C), and the score is 15% (Figure 3E). When bioactive fractions were co-administered with *S*. Heidelberg, less neutrophil infiltration was detected (Figure 3D) and the score significantly decreased to 1% as the controls (Figure 3E). Our data suggest that *S*. Heidelberg activated strong recruitment of inflammatory cells restored by the treatment with the bioactive fractions (Figure 3E).

**Figure 4:**
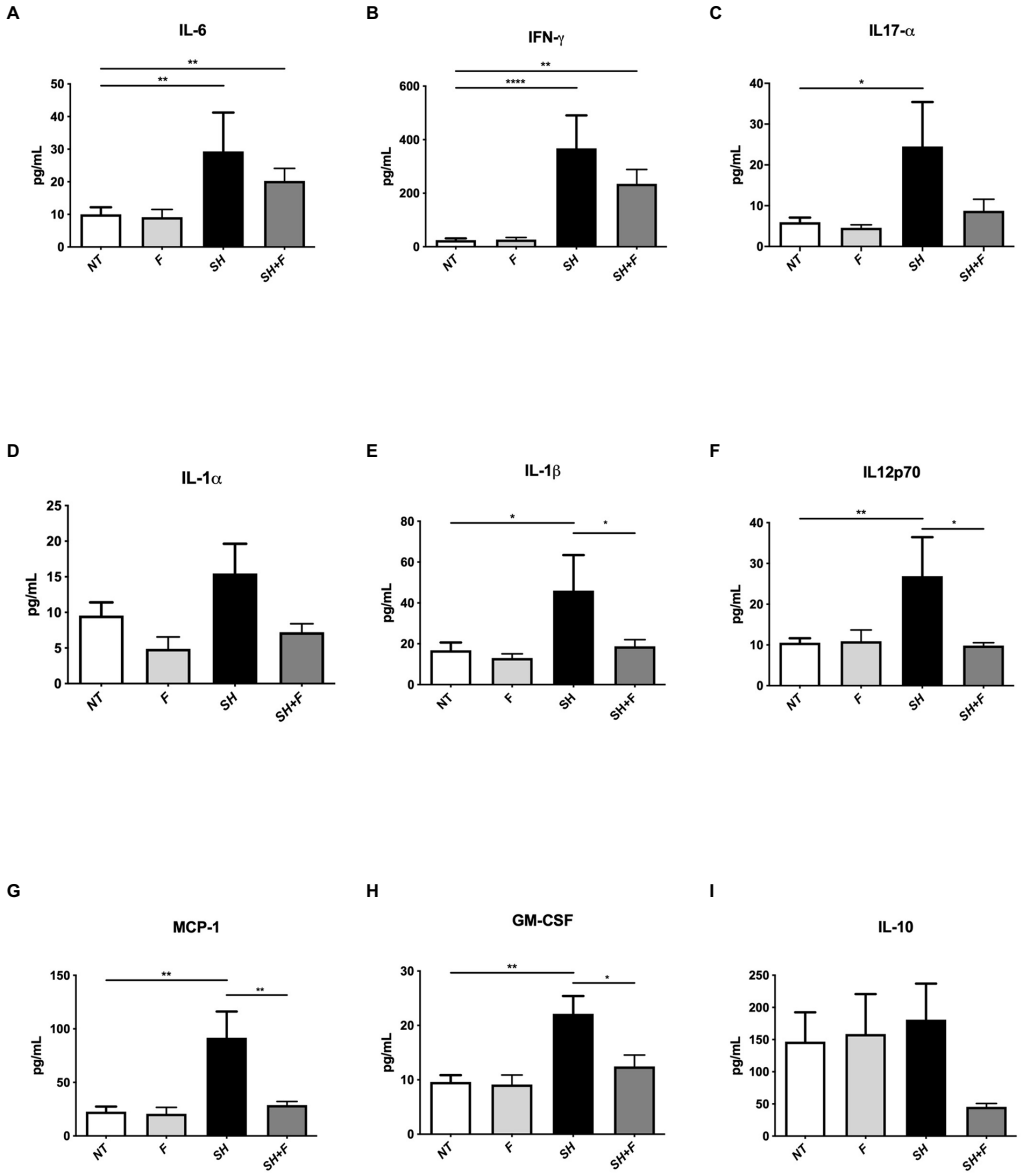
Bioactive fractions attenuated systemic cytokines during *Salmonella* Heidelberg infection. The concentration of inflammatory cytokines in mice plasma was measured using a Legendplex mouse inflammatory panel composed of a magnetic bead panel. The concentration of IL-6 **(A)**, IFN-γ **(B)**, IL17-α **(C)**, IL-1α **(D)**, IL-1β **(E)**, IL12p70 **(F)**, MCP-1 **(G)**, GM-CSF **(H)**, and IL-10 **(I)** are indicated. Normality of data was verified by Shapiro test. ANOVA one way was used for *n* = 5. All data were presented as means (SEM). *p < 0.05; **p < 0.01; ***p < 0.001; ****p < 0.0001.

Because the fractions decreased infiltration of inflammatory cells, we further evaluated cytokine levels in these mice. We selected and examined the levels of pro-inflammatory and anti-inflammatory cytokines in the mice plasma. The results showed a significant increase in the pro-inflammatory cytokines IL-1β, IL-6, IFN-γ, and IL12p70 in the group infected with *S*. Heidelberg (Figures 4A,B,E,F). IL-17α, MCP-1, and Granulocyte-macrophage colony-stimulating factor (GM-CSF), mediators implicated in the recruitment and differentiation of a variety of immune/inflammatory cells, were also increased, which correlated with neutrophils infiltration in the colon of these mice (Figures 4C,G,H).

The addition of bioactive fractions significantly reduced circulating levels of chemoattractant and inflammatory cytokines, which are elevated in *S*. Heidelberg infection. These results and those obtained with H&E staining suggested that a key aspect of bioactive fractions action might include modulation of inflammatory cells mobilization. IL-10 is decreased by 3-fold in mice receiving *Salmonella* and bioactive fractions; however, the difference was not significant (Figure 4I).

### Bioactive fractions improved tight junction genes expression in mice colon

**Figure 5:**
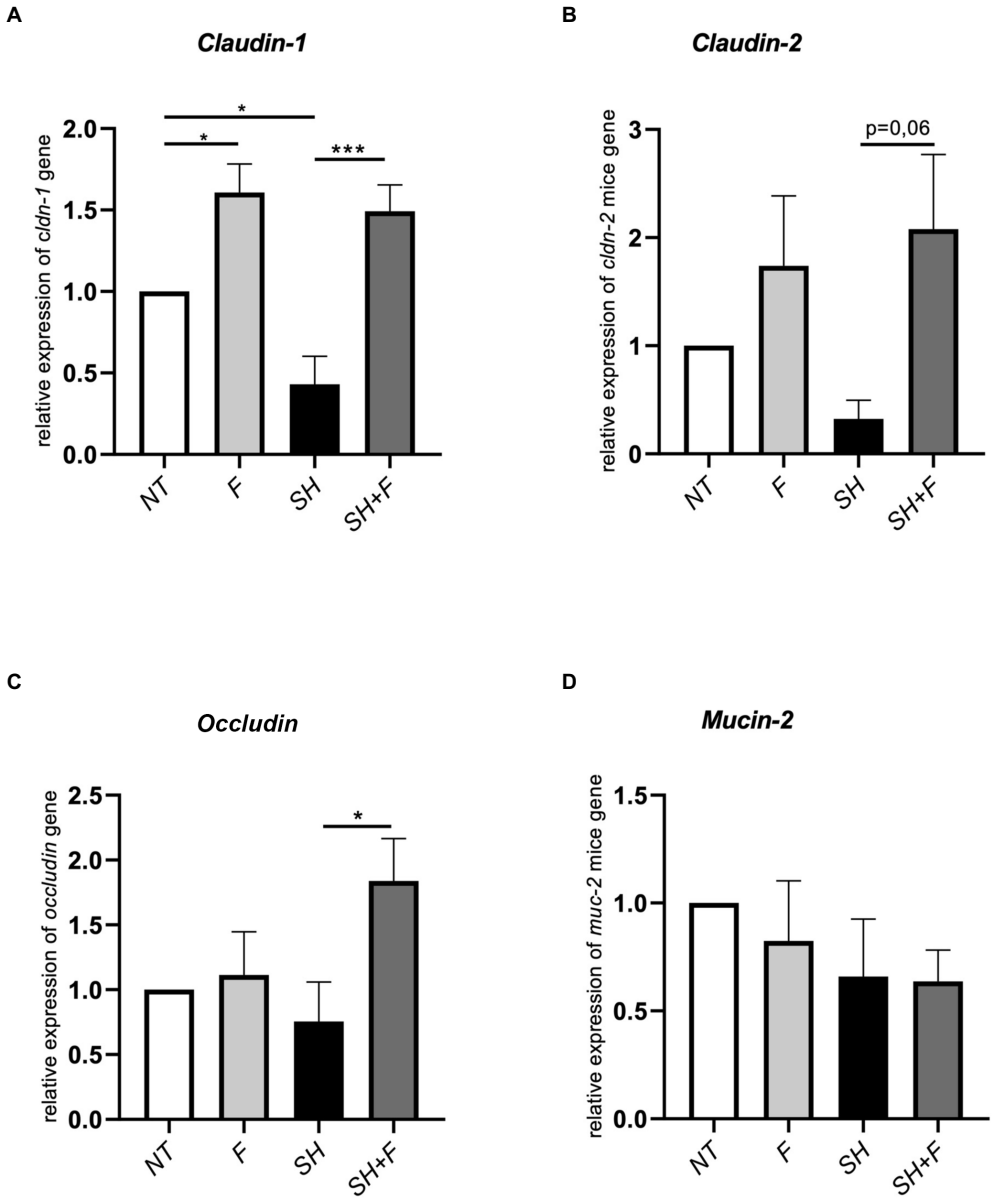
Bioactive fractions improved tight junction genes expression in mice colon. Relative expression of **(A)** claudin-1 (*cldn-1*), **(B)** *claudin-2* (*cldn-2*), **(C)** *occludin*, and **(D)** *mucin-2* (*muc-2*) in colon tissue from mice treated with bioactive fractions **(F)**, mice infected with *S*. Heidelberg (SH) and mice receiving both *S*. Heidelberg and bioactive fractions (SH + F). Results are normalized to control mice without fraction and without *Salmonella* (NT). All genes were reported based on the reference housekeeping gene *hprt* (*hypoxanthine phosphoribosyltransferase 1*). Normality of data was verified by Shapiro test and ANOVA one way was used. All data were presented as means (SEM), *n* = 4–5. *p < 0.05 and ***p < 0.001.

Tight junction genes [claudin (*cldn-1*, *cldn-2)* and *occludin*] expression was downregulated after *S*. Heidelberg infection with a significant difference for *cldn-1* (Figures 5A–C). However, bioactive fractions co-administration with *S*. Heidelberg significantly reversed the expression of *cldn-1* (Figure 5A) and *occludin* (Figure 5C). *Cldn-1* was also upregulated in mice receiving only fractions without *Salmonella*, contrary to *cldn-2*, and *occludin*. When the impact of fractions on the mucus layer was evaluated by detecting *mucin*-2 gene (*muc*-2), no significant difference was detected in *muc*-2 expression (Figure 5D). The bioactive fractions are beneficial to maintaining barrier function through upregulating the relative RNA abundance of *occludin* and *cldn-1*.

### Bioactive fractions decreased the genus *Alistipes* without altering gut microbiota diversities

**Figure 6:**
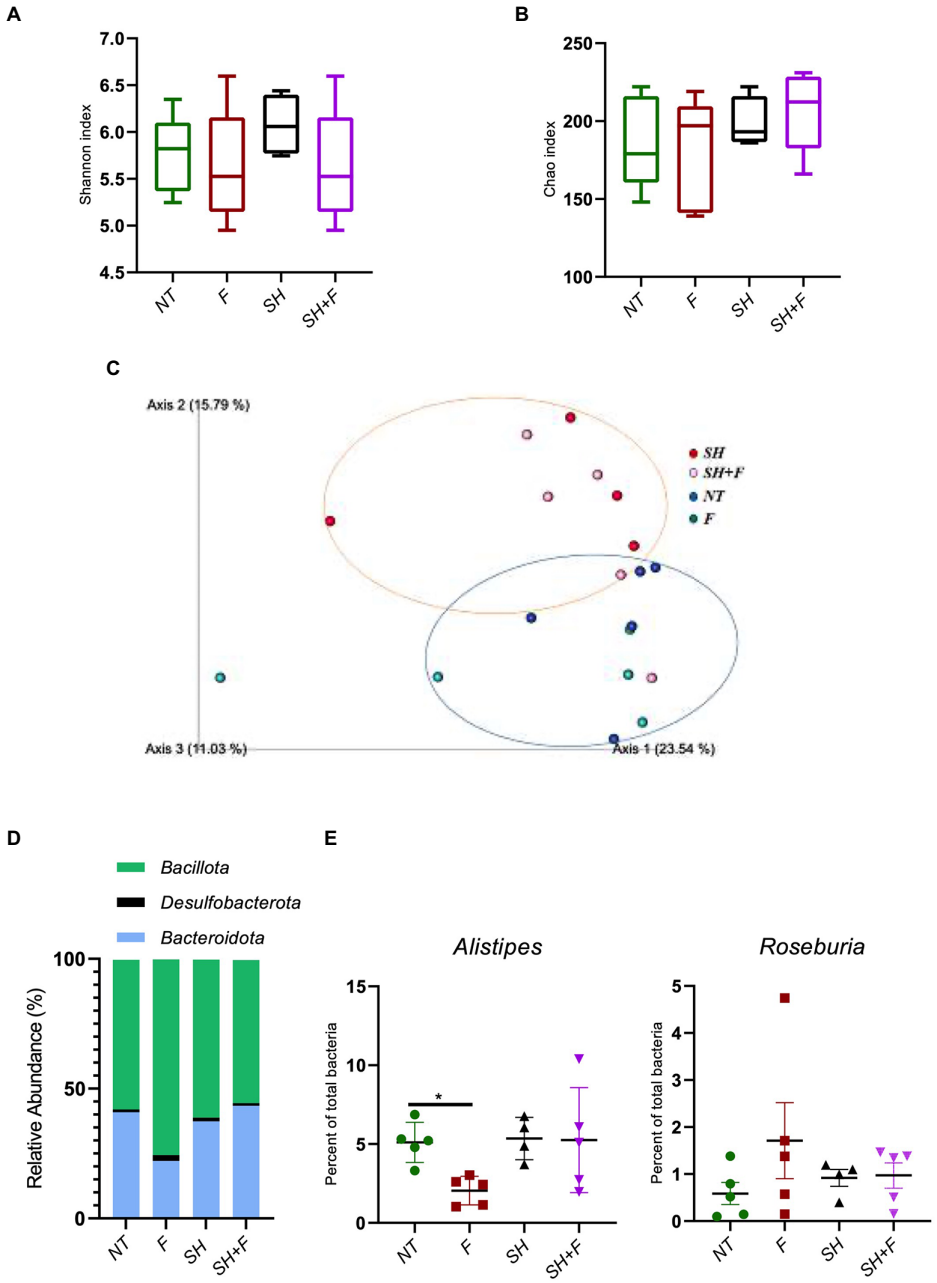
Bioactive fractions decreased the genus *Alistipes* without modifying gut microbiota diversities. Studies on the mice microbiota (16S analysis) were performed on fecal samples. Alpha and Beta diversity studies were completed using a script compatible with the Qiime 2 visualization software. Alpha diversity was represented by the Shannon index **(A)** and the Chao index **(B)**. **(C)** Inter-individual Beta diversity was represented by Qiime 2 visualization based on beta diversity distance (Bray–Curtis dissimilarity). The blue circle represented the Beta diversity cluster of the mice groups not infected with *S*. Heidelberg. The orange circle represented the Beta diversity cluster of the groups of mice infected with *S*. Heidelberg. **(D)** Relative abundance of bacteria phyla was represented excepted phyla with a relative abundance of <0.5%. **(E)** Quantification of *Alistipes* and *Roseburia* genera in each mice group.

The alpha diversity showed no significant difference between the four mice groups (Figures 6A,B). However, the beta diversity was significantly different (value of *p* = 0.003) between mice receiving *S*. Heidelberg and mice without *S*. Heidelberg, suggesting a change in diversity caused by *S*. Heidelberg infection (Figure 6C). Bioactive fractions administration alone or with *S*. Heidelberg did not impact the beta diversity of mice gut microbiota. At the phylum level, the compositional analytic results showed that *Bacillota*, *Bacteroidota*, and *Desulfobacterota* were the most dominant bacteria in all groups, with no significant variation was detected between groups (Figure 6D). Nevertheless, the abundance of the phylum *Bacteroidota* was not significantly declined in the group receiving the fractions alone compared to the other groups. However, at the genus level, *Alistipes* were significantly decreased in the group receiving the bioactive fractions alone, whereas in the presence of *S*. Heidelberg, no significant decrease was induced (Figure 6E). On the other hand, when we analyzed the genus *Roseburia*, described as decreasing inflammation (Tamanai-Shacoori et al., 2017; Nie et al., 2021), the difference was not significant (Figure 6E).

### Identification of potential bioactive compounds in fractions: cholic acid and deoxycholic acid

**Figure 7:**
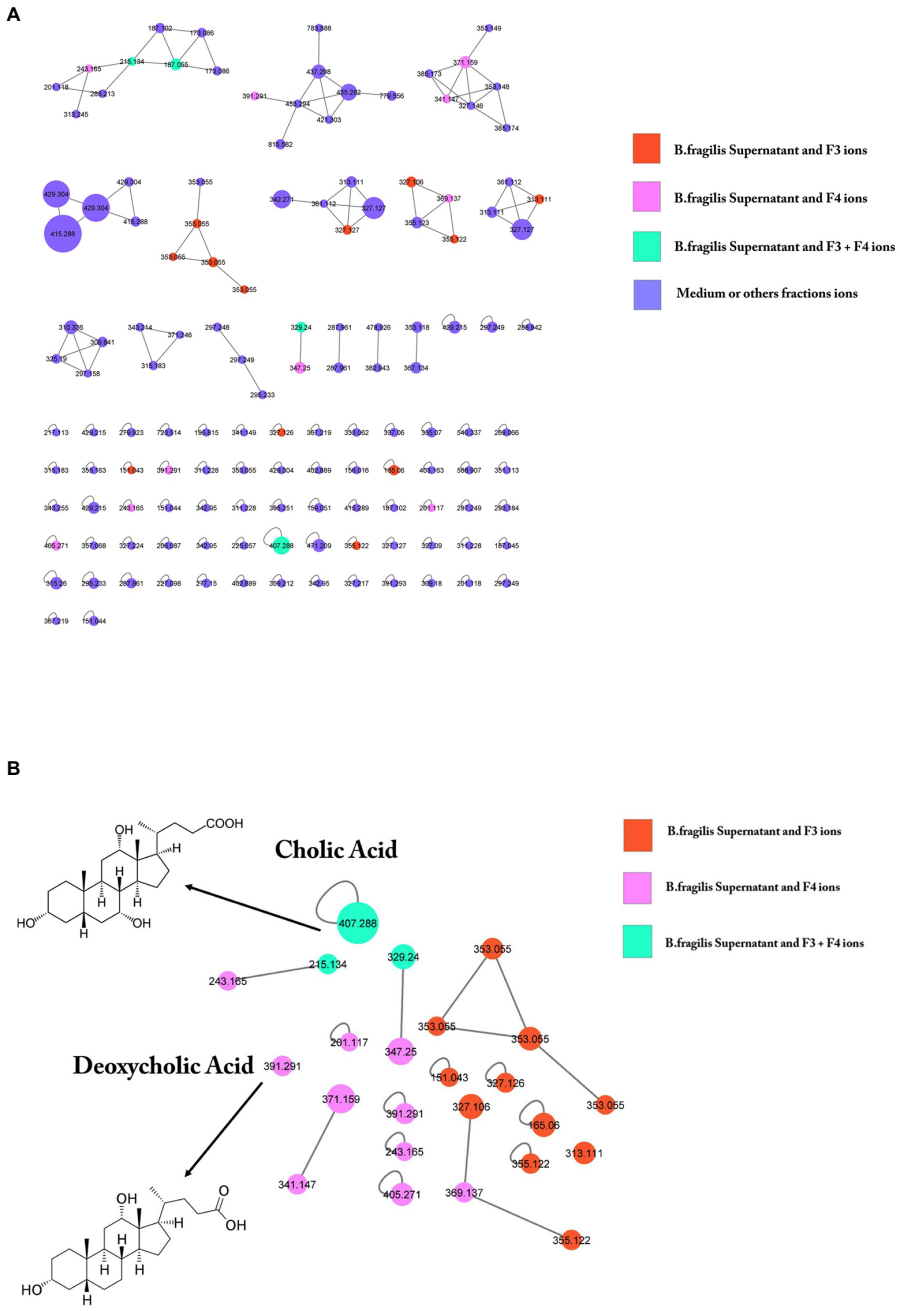
GNPS molecular networking of negative ion MS/MS spectral data showing a selection of ions in bioactive fractions (F3 and F4). **(A)** Data were recorded in negative ionization mode. The nodes display the measured average masses of the molecular ions with identical MS/MS spectra. The numbers on the nodes represent the parent mass of the molecules. Node size reflects the bioactivity scores of the ions; node color indicates the abundance in the different conditions. The nodes in orange correspond to compounds present both in *B. fragilis* supernatant and in F3; nodes in pink correspond to compounds present both in supernatant and F4; nodes in green correspond to compounds present in all (supernatant, F3 and F4); nodes in purple correspond to ions solely found in DMEM. GNPS analysis of *B. fragilis* supernatant and bioactive fractions after LC–MS/MS showed the presence of 24 specific molecules of these fractions, 11 molecules for F3, 10 molecules for F4, and 3 molecules for both. **(B)** Among them, cholic acid (CA) and deoxycholic acid were identified.

To identify candidate bioactive molecules present in the fractions inhibiting *S*. Heidelberg translocation, HPLC-HRMS^2^ profiles were acquired (in both positive and negative polarities). These data were subsequently processed using the classical molecular-networking workflow (Wang et al., 2016; Figure 7A). Only ions specific to the supernatant (SN) and bioactive fractions were investigated; other contents of the culture medium (complete DMEM) and the other fractions (F1, F2a, F2b, and F5) were not selected for further processing. The cursory examination of the molecular network revealed 24 ions specific to the bioactive fractions F3 and F4. Among them, we focused on two metabolites identified by the GNPS database, including CA and DCA (Figure 7B), described as potential inhibitors of pathogenic bacteria (Schmidt et al., 2001). The prominent node of CA (*m/z* 407.288) indicated that CA is a major compound in the supernatant and the two associated bioactive fractions (F3 and F4). DCA was only present in the supernatant and the fraction F4. The unidentified ions could correspond to either new molecules or to compounds that have not yet been uploaded to the GNPS repositories. Two potential candidate ions (*m/z* 329.24 and *m/z* 215.134), not recognized by comparison against the GNPS spectral libraries, were also found in the supernatant, in F3, and F4 (green circle). Figure 7B also showed that F3 contained 11 unidentified molecules (orange circle) whereas F4 contained 10 specific metabolites (pink circle).

Then, HPLC coupled with an ELSD detector or mass spectrometric detection was used to quantify CA and DCA in the supernatant and fractions. Calibration curves of the synthetic reference compounds CA and DCA were used to estimate their concentrations. CA was detected at 156 μmol/l in the supernatant, at 36.7 and 19.6 μmol/l in F3 and F4, respectively, whereas DCA was not detected, probably due to it occurring below the detection limit (Table 2).

**Table 2:** Cholic acid (CA) quantification in supernatant (SN) and in bioactive fractions (F3, F4) after HPLC-ELSD (High-performance liquid chromatography- evaporative light scattering detector) analysis.

*Bacteroides fragilis* derivate	CA (mg/ml)	CA (μmol/l)
SN	0.064	156.0
F3	0.015	36.7
F4	0.008	19.6

**Figure 8:**
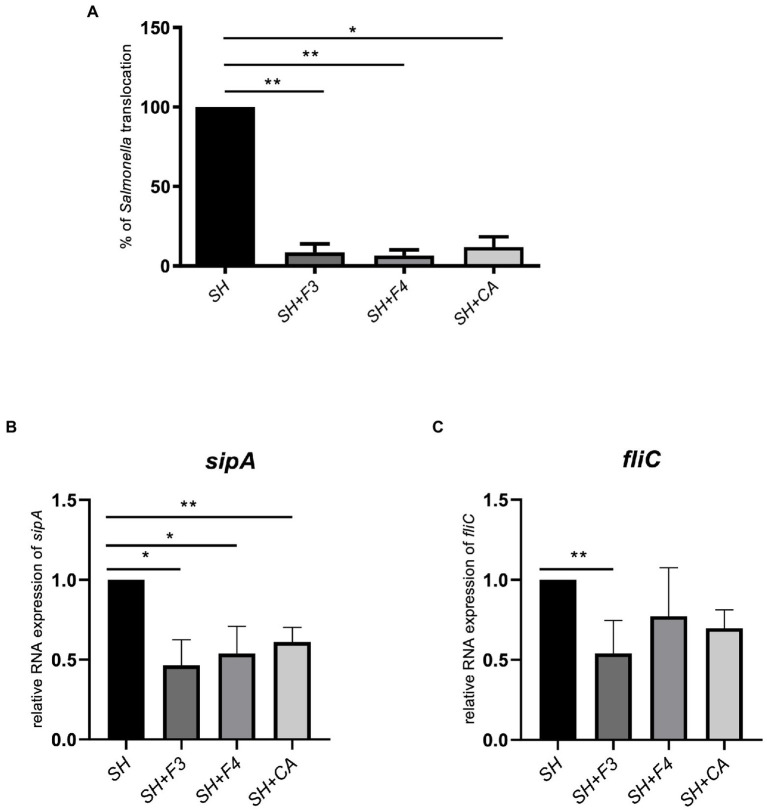
CA identified in bioactive fractions inhibited *S*. Heidelberg translocation *in vitro*. **(A)** Synthetic CA, at 156 μmol/l, corresponding to the concentration found in bioactive fractions, inhibited *Salmonella* translocation compared to fractions F3 and F4 in the multicellular model. **(B)** CA downregulated only *sipA* expression but not *fliC* **(C)**. All results were normalized to the control *Salmonella* alone. All genes were reported based on the reference housekeeping gene *16S* RNA. Shapiro test was performed to verify normality and one-way ANOVA test was performed on *n* = 5. All data were presented as means (SEM). *p < 0.05; **p < 0.01.

This high level of CA in the supernatant confirmed the results obtained in the molecular network, leading us to investigate the role of CA in *S*. Heidelberg translocation inhibition. We have incubated *S*. Heidelberg with 156 μmol/L of synthetic CA on the tri-cellular model, under the same conditions as described above. The data showed that CA significantly inhibited the translocation of *S*. Heidelberg, and decreased the expression of *sipA* as F3 and F4 fractions (Figures 8A,B). However, for *fliC* expression in the presence of CA, the difference was not significant (*p* = 0.10). F3 downregulated both *sipA* and *fliC*, seeming more efficient than CA and F4. In addition, F3 showed 11 unidentified ions (Figure 7B in orange), suggesting that one (or more) of these metabolites could play a crucial role in *S*. Heidelberg virulence expression. It will be indispensable to identify them to characterize their molecular mechanisms.

## Discussion

Gut microbiota contains healthy bacteria producing molecules that could help to fight diseases in the gut and other organs (Antunes et al., 2014; Postler and Ghosh, 2017; Sittipo et al., 2019). SCFAs, particularly butyrate and propionate, reduced inflammatory bowel diseases (IBD) and decreased inflammation in patients (Silva et al., 2018; Li et al., 2021). These compounds were shown to increase the expression of cell junction genes and to decrease that of pro-inflammatory cytokines (IL-1β, IL-6, IL-8, TNF-α) in the gut and in other organs (Huang et al., 2021; Peña-Rodríguez et al., 2022; Yue et al., 2022). Desaminotyrosine, produced by *Clostridium orbiscidens*, can protect against influenza virus infection by increasing the expression of IFN-I way (Steed et al., 2017; Wei et al., 2020). Other bacterial metabolites, BAs, have emerged as key signaling molecules, which participate in multiple metabolic and inflammatory pathways (Fiorucci et al., 2021; Thomas et al., 2022). BAs are the end-product of cholesterol metabolism generated in the liver by a chain of enzymatic reactions (Cai et al., 2022). These hepatic pathways generate mainly two primary BAs, i.e., CA and chenodeoxycholic acid (CDCA). These molecules are conjugated by taurine or glycine in the liver to form taurocholic acid (TCA) and glycocholic acid (GCA; Yang et al., 2021). In the colon, the unconjugated BAs are further transformed into secondary BAs, DCA, and lithocholic acid (LCA) *via* 7*α*-dehydroxylation.

It is known that an intact gut microbiota protects the host against infection by gastrointestinal pathogens by colonization resistance, by competition for nutrients and host receptors and by secretion of antimicrobial substances (Yurist-Doutsch et al., 2014; Vogt et al., 2015; Peng et al., 2022). We have previously shown that the cell-free supernatant of *B. fragilis* inhibited the translocation of *S*. Heidelberg (Vernay et al., 2020). To better characterize the molecules present in this supernatant, we fractionated it by HPLC. Two fractions, F3 and F4, could inhibit S. Heidelberg translocation in a model mimicking an intestinal epithelium previously developed (Vernay et al., 2020) while they did not interfere with the growth of *S*. Heidelberg. Conversely, these bioactive fractions affected the expression of virulence-associated genes in this *S*. Heidelberg. SipA effector proteins are required for membrane ruffling and invasion of *Salmonella* into the host cell. Meanwhile, flagella (FliC) trigger rapid and efficient contact with the cells lining the epithelium, allowing *Salmonella* to penetrate the gastrointestinal mucus layer. As FliC and SipA play a key role in *Salmonella* pathogenesis, we have investigated the impact of F3 and F4 on their expression. We have shown that *sipA* was significantly inhibited in the presence of F3 and F4 whereas *fliC* was only significantly inhibited with F3. Besides, F5 which did not inhibit *Salmonella* translocation had no impact on *SipA* and *fliC*expression. A difference was detected in *fliC* expression between F3 and supernatant, suggesting that the bioactive molecules in F3 could be inactivated when they are in the supernatant probably due to the presence of other compounds. It has been shown in a study that the metabolite Bac70 purified from *Bacillus atrophaeus* supernatant has a better antimicrobial activity than supernatant itself (Sarjoughian et al., 2020). Several studies have shown that some strains of *Lactobacillus* and *Bifidobacterium* interfere with the ability of *Salmonella* Typhimurium to adhere to host cells (Yang et al., 2014; van Zyl et al., 2020; Kim et al., 2021). These probiotics produce compounds that do not have a direct bactericidal effect but contribute to anti-infectious activities by inhibiting the binding of pathogenic bacteria to the mucosal surface. Durant et al., 2000 showed that lactic acid affected the expression of the *hilA* and *invF* virulence factors of *Salmonella* (Durant et al., 2000). Liévin-Le Moal et al., 2011 demonstrated that *L. acidophilus* and its secreted products inhibited the entry of *S. enterica* serovar Typhimurium into human intestinal Caco-2 cells by disrupting the swimming motility of this pathogen (Liévin-Le Moal et al., 2011).

To demonstrate that the antagonistic activity of bioactive fractions against *S*. Heidelberg observed *in vitro* could also occur *in vivo*, *S*. Heidelberg was administered orally to mice. The infected mice receiving also bioactive fractions (mixture of F3 and F4) exhibited a significantly decrease of viable *S*. Heidelberg level in Peyer’s Patches and spleen, showing that the bioactive fractions inhibited the spread (translocation) of *S. Heidelberg in mice*. Besides, the number of bacteria was not significantly different between groups receiving *Salmonella* alone or with fractions in feces and colon. The presence of *Salmonella* in the feces after treatment with the fractions showed that these fractions did not inhibit *Salmonella* growth but its translocation since no bacteria are present in the Peyer’s patches. Taken together, these *in vivo* data reinforced the *in vitro* results concerning the effect on translocation and not on *Salmonella* growth. In the presence of F3 and F4 fractions, *fliC* expression was downregulated. It is possible that the inhibition of *S*. Heidelberg motility delayed its translocation of *S*. Heidelberg across the intestinal barrier, thus maintaining *Salmonella* in gut lumen which is eliminated in feces.

In addition, these bioactive fractions are also associated with a decrease in inflammation, as shown by a reduced rate of neutrophils infiltration and a decrease in systemic cytokine concentrations. Neutrophils infiltrations, in mice receiving both *S*. Heidelberg and bioactive fractions are related to a decreased expression of GM-CSF and MCP-1, involved in the recruitment, differentiation, and proliferation of several immune cells (Bayoumi and Griffiths, 2010). Moreover, bioactive fractions attenuated the severity of *S*. Heidelberg infection in mice by decreasing the production of IL-1β and IL12p70. Several studies have indicated that *B. fragilis* and its polysaccharide A (PSA) can alleviate intestinal inflammation in an animal model of colitis and confer protection against pathogens such as *Mycobacterium tuberculosis* and *Clostridium difficile* (Deng et al., 2018; Eribo et al., 2022). This beneficial effect may be associated with the production of IL-10, as shown in colitis and viral infection (Cohen-Poradosu et al., 2011; Blandford et al., 2019; Ramakrishna et al., 2019). However, in our study, IL-10 was decreased but not significantly when *Salmonella* was combined with bioactive fractions. Several studies described that *Salmonella* could use IL-10 to disseminate in several organs. Thus, the decrease of IL-10 in the presence of the bioactive fractions may inhibit it, supporting our results concerning *S*. Heidelberg enumeration in spleen and Peyer’s patches (Salazar et al., 2017). Besides, several studies suggested that commensal-derived extracellular products, in particular (PSA)-containing OMVs, isolated from *B. fragilis*, regularly maintain intestinal homeostasis, they are also known to have altered functions during infection and diseases to ensure host survival (Chu et al., 2016; Durant et al., 2020; Engevik et al., 2021; Gul et al., 2022). We can speculate that fractions, in the infection with pathogenic bacteria, can have altered role in IL-10 regulation. Other probiotic strains such as *Lactobacillus* and *Bifidobacterium* also decreased inflammation *in vivo*. Sagheddu et al., 2020 and Jenab et al., 2020 indicated that natural compounds of these strains decreased inflammation (Jenab et al., 2020; Sagheddu et al., 2020).

By investigating gut microbiota in mice, we have shown that administration of *S*. Heidelberg alters the diversity of the microbiota as described in several studies, but that administration of bioactive fractions does not cause an alteration in the alpha and beta intestinal diversities (Khan and Chousalkar, 2020). The results suggested that bioactive fractions only target the virulence of *S*. Heidelberg without disrupting other gut phyla. Nevertheless, we have demonstrated that at the genus level, *Alistipes* were decreased in the presence of the bioactive fractions whereas *Roseburia*, known to have anti-inflammatory properties (Tamanai-Shacoori et al., 2017), was not significantly decreased. *Alistipes* spp. are described in dysbiosis associated with increased intestinal inflammation and depression (Parker et al., 2020; Wu et al., 2021; Zheng et al., 2021). The *Alistipes* genus is a well-known stress target in rodents and humans (Li et al., 2017). Recent studies have shown that lipopolysaccharides (LPS) of *Alistipes* can be pro-inflammatory (Parker et al., 2020). However, the *Alistipes* genus includes several species with anti-inflammatory properties by producing sulfonolipids (SLs), a type of sphingolipid that has anti-inflammatory effects by suppressing the activation of cytokines such as TNF-α in mice (Walker et al., 2017). The decrease of *Alistipes* abundance in mice that received the fractions can explain the anti-inflammatory effects of the bioactive fractions (as indicated by IL1-β decrease, Figure 4) or the anti-stress effect, which needs to be investigated. As these bioactive fractions do not significantly modify the fecal microbiota diversities, they can be considered to treat *Salmonella* infections without damaging the commensal flora in contrary to the conventional antibiotic treatment or approach (Ogawa et al., 2020; Ramirez et al., 2020).

In this study, in mice receiving both bioactive fractions of *B. fragilis* and *Salmonella*, the expression of *cldn-*1 and *occludin* in the colon was significantly increased compared to *Salmonella* alone, suggesting an improvement in the intestinal barrier. In the presence of the bioactive fractions alone, only *cldn-1* was upregulated. It was already described that *B. fragilis* NTBF ZY-312 induced *occludin* over-expression in mice with AAD (Antibiotics Associated Diarrhea). In addition, Wang et al., 2021 showed that *B. fragilis* NTCC 9343 increased the expression of the tight-junction proteins ZO-1, occludin and claudin-1 in the colon against DSS-induced ulcerative colitis, whereas the *B. fragilis* strain FJSWX11BF played no protective role (Wang et al., 2021). However, in both of these studies, the bacterial compounds implicated in this regulation were not identified as they used only live bacteria.

To identify *B. fragilis* metabolites contributing to the improvement of host health, we have used LC–MS/MS and molecular networking analysis. Our data indicated that these bioactive fractions of *B. fragilis* contained identified metabolites, CA and DCA, among many other compounds that were not identified by comparison against GNPS spectral libraries. In the intestinal tract, numerous gut bacteria exhibit bile salt hydrolase (BSH) enzymatic activity responsible for bile acid (BA) deconjugation, and subsequently forming secondary BAs metabolites, such as DCAs (Gérard et al., 2007; Yang et al., 2021; Guo et al., 2022). BSH has been identified in extensive bacterial genera, including *Lactobacillus*, *Bifidobacterium*, *Clostridium*, *Bacteroides* and *Enterococcus* (Song et al., 2019). The BSH enzyme was identified in the *B. fragilis* ATCC 25285 strain that we have used in this study by analyzing its genome (NCBI accession: NC_003228.3) with the Genious software (data not shown). *Bacteroides fragilis* can deconjugate the bile salts GCA, TCA to release CA but also the bile salts glycodeoxycholic acid (GDCA) and taurodeoxycholic acid (TCDA) to produce DCA. After quantifying each bioactive fraction, CA was tested *in vitro* for its ability to inhibit *S.* Heidelberg translocation. As the bioactive fractions, CA can reduce virulence by inhibiting *sipA* but not *fliC* expression. Sievers et al., 2019 showed that CA did not decrease *fliC* in *Clostridium difficile* infection in contrast to DCA (Sievers et al., 2019). Eade et al., 2016 indicated that bile acids comprising CA and DCA directly impact *sipC*, *sopB*, and *hilD* expression with a synergic effect of CA and DCA administration (Eade et al., 2016). Several studies demonstrated that CA is more active when conjugated to *N*-methyl benzimidazole or with alkyl chains of varied hydrophobicity (Yadav et al., 2019; Kakkar et al., 2022). Besides, as DCA could not be quantified in our study, we can speculate that other molecule (s) not identified in the database could downregulate *fliC* (Sievers et al., 2019; Metzendorf et al., 2022).

However, this study had some limitations. Experiments were performed in laboratory conditions, which did not fully reflect the complexity of the human gut and the environment that a commensal bacterium may have to play its beneficial role. Moreover, more studies on complete DMEM containing 20% of serum are required to understand the mechanisms implicated in CA formation in the presence of *B. fragilis*. Besides using mice model can be considered as the other limitation of the study. Mouse and human are quite similar in physiology and anatomical structures, and this is one of the reasons why mouse models have been widely used in biomedical studies. However, the anatomy of the mouse and human intestinal tract also have prominent differences, which might be shaped by their diverging diets, feeding patterns, body sizes and metabolic requirements (Nguyen et al., 2015; Xiao et al., 2015). Overall, the gut microbiota of human and mice are dominated by two major phyla, *Bacteroidota* and *Bacillota* and 85% of bacterial genera found in the mouse gut microbiota are not present in human (Ley et al., 2005).

To conclude, we showed for the first time that *B. fragilis NTBF*, ATCC 25285, produced CA and unidentified molecule(s) inhibiting *Salmonella* virulence factors expression such as *fliC* and *sipA* belonging to the T3SS system. These potential molecules prevent the dissemination of *Salmonella* in target organs such as the spleen and Peyer’s patches. The use of bioactive fractions to target the T3SS system will reduce the virulence of *Salmonella* without inducing stress on the bacteria and thus will limit the emergence of antibiotic resistance. Therefore, targeting the T3SS system with bioactive fractions can be an alternative approach to fighting pathogenic bacteria.

## Data availability statement

The datasets presented in this study can be found in online repositories. The names of the repository/repositories and accession number(s) can be found at: NCBI—PRJNA876074.

## Ethics statement

All experimental protocols were approved by the Adaptive Therapeutics Animal Care and Use Committee (APAFIS#31484–2,021,050,308,355,787 v6).

## Author contributions

OL, ST, and LB conceptualized and designed the study. TG, NO, SF, PP, AS, PL, IC, SG, and SD performed experiments and acquired data. TG and LB analyzed and interpreted the data; drafted the manuscript and created figures; and edited the manuscript. AS participated in English correction. All authors contributed to the article and approved the submitted version.

## Funding

This work was supported by the French National Institute for Health and Medical Research (Inserm-Transfert, Preuve de Concept) with two grants N°R19053NS in 2019 and N° R21001NS in 2020.

## Conflict of interest

The authors declare that the research was conducted in the absence of any commercial or financial relationships that could be construed as a potential conflict of interest.

## Publisher’s note

All claims expressed in this article are solely those of the authors and do not necessarily represent those of their affiliated organizations, or those of the publisher, the editors and the reviewers. Any product that may be evaluated in this article, or claim that may be made by its manufacturer, is not guaranteed or endorsed by the publisher.

## Footnotes

^1^ https://qiime2.org/

^2^ http://gnps.ucsd.edu
